# Cdx1 and Cdx2 Exhibit Transcriptional Specificity in the Intestine

**DOI:** 10.1371/journal.pone.0054757

**Published:** 2013-01-30

**Authors:** Stephanie Grainger, Alexa Hryniuk, David Lohnes

**Affiliations:** Department of Cellular and Molecular Medicine, University of Ottawa, Ottawa, Ontario, Canada; National Cancer Institute, United States of America

## Abstract

The caudal-related homeodomain transcription factors Cdx1 and Cdx2 are expressed in the developing endoderm with expression persisting into adulthood. *Cdx1^−/−^* mutants are viable and fertile and display no overt intestinal phenotype. *Cdx2* null mutants are peri-implantation lethal; however, conditional mutation approaches have revealed that Cdx2 is required for patterning the intestinal epithelium and specification of the colon. Cdx2 is also necessary for homeostasis of the intestinal tract in the adult, where Cdx1 and Cdx2 appear to functionally overlap in the distal colon, but not during intestinal development. Cdx1 and Cdx2 exhibit complete overlap of expression in the intestine, although they differ in their relative levels, with Cdx1 maximal in the distal colon and Cdx2 peaking in the proximal cecum. Moreover, Cdx1 protein is graded along the crypt-villus axis, being abundant in the crypts and diminishing towards the villi. Cdx2 is expressed uniformly along this axis, but is differentially phosphorylated; the functional relevance of these expression domains and phosphorylation is currently unknown. Cdx1 and Cdx2 have been suggested to exhibit functional specificity in the intestinal tract. In the present study, using cell-based models, we found that relative to Cdx1, Cdx2 was significantly less potent at effecting a transcriptional response from the *Cdx1* promoter, a known Cdx target gene. We subsequently assessed this relationship *in vivo* using a “gene swap” approach and found that Cdx2 cannot substitute for Cdx1 in this autoregulatory loop. This is in marked contrast with the ability of Cdx2 to support Cdx1 expression and function in paraxial mesoderm and vertebral patterning, thus providing novel *in vivo* evidence of context-dependent transcriptional specificity between these transcription factors.

## Introduction

The intestinal tract is derived primarily from definitive endoderm, formed as epiblast cells ingress through the primitive streak, with some contribution from visceral endoderm [3]. The gut is subsequently patterned in an anterior to posterior order, which is reflected by the distinct functionalities of the esophagus, stomach, small and large intestines and associated accessory organs [4]. The small intestine is a highly specialized structure characterized by the finger-like villus projections and invaginating crypts which together comprise the crypt-villus axis. A pool of stem cells is housed in the base of the crypt region [5], [6] which divide to produce highly proliferative transit-amplifying (TA) cells. These subsequently differentiate into enterocytes, Goblet cells, and enteroendocrine cells which migrate towards the tip of the villus and are shed 5–7 days later in the mouse. A fourth TA cell derivative, the Paneth cell, migrates to the base of the crypt and reside there with a lifespan of approximately 28 days. The colon lacks villi, which are replaced with a flattened epithelium which harbors mostly colonocytes and Goblet cells [4].

While the molecular mechanisms governing intestinal patterning are incompletely understood, the Cdx gene products are known to play an important role in this process [7], [8], [9], [10]. Cdx1, Cdx2 and Cdx4 are homeodomain transcription factors related to *caudal* in *Drosophila*. Cdx1 and Cdx2 are expressed in the developing endoderm, where their expression persists in the intestine throughout life [1], [2], [11], [12]. *Cdx1^−/−^* mutants are viable and fertile and exhibit vertebral homeotic transformations, but no overt intestinal phenotype [13]. *Cdx2^−/−^* mutants are peri-implantation lethal [11], [14], however conditional deletion strategies have revealed key roles for Cdx2 in diverse processes, including axial elongation and mesoderm patterning [15], [16], [17] and in the definitive endoderm and intestinal epithelium [7], [8], [9].

Although poorly conserved outside of the homeodomain, considerable evidence suggests that the Cdx proteins functionally overlap in several developmental processes including neural tube closure, axial elongation and mesodermal patterning [15], [18], [19], [20]. This is consistent with gene substitution approaches that have shown that Cdx2 can replace Cdx1 in vertebral patterning [21]. However, the functional relatedness between Cdx1 and Cdx2 in the intestine has not been thoroughly investigated *in vivo*.

Cdx1 and Cdx2 are differentially expressed in the intestinal epithelium, with Cdx1 highest in the distal colon and Cdx2 maximal in the cecum. Furthermore, Cdx1 expression is graded along the crypt-villus axis, with more abundant levels in the crypts relative to the villi, while Cdx2 is expressed uniformly along this axis, but is differentially phosphorylated [1], [2]. Although Cdx1 appears dispensable for development of the developing small intestine [7], [13], [22], Cdx1, together with Cdx2, may play a role in specification of the colon [9]. Furthermore, in the adult, Cdx1 functionally overlaps with Cdx2 in regulating intestinal homeostasis and colon patterning [8], [23]. In contrast to these observations, a number of studies in tissue culture models suggest that Cdx1 and Cdx2 exhibit specificity in the intestine. For example, the Cdx target *apical sodium-dependent bile acid transporter (ASBT)* is preferentially regulated by Cdx2 [24]. Furthermore, the calcium channel *MS4AI2* is responsive to Cdx2, but not Cdx1 [25], while the *intestinal alkaline phosphatase* gene is activated by Cdx1 and inhibited by Cdx2 [26]. Conversely, a number of intestinal genes have been reported to respond similarly to Cdx members in tissue culture models, such as *SLC5A8* [27]. Functional equivalence between Cdx members is further exemplified by loss of expression of many intestine-specific genes such as *Treh*, *Lct* and *Heph* in *Cdx2* ^−/−^ and *Cdx1* ^−/−^ *Cdx2^−/−^* mutants, while other genes, such as *Slc7a8* and *Alpi,* appear to exhibit Cdx-type specific response [8].

The above observations suggest that Cdx1 and Cdx2 may be functionally distinct in certain contexts. To examine this further, we assessed regulation of the *Cdx1* promoter, which is a Cdx1 target gene involved in an autoregulatory loop [28]. Using tissue culture models, we found that Cdx2 is significantly less potent compared to Cdx1 on this promoter, and that this difference can be mapped to differences in N-terminal transactivation sequences. To test this interaction *in vivo*, we examined mice in which Cdx2 had been substituted for Cdx1 (termed *Cdx1^2ki/2ki^* hereafter) [21] and lacking endogenous *Cdx2*. Using this model, we find that Cdx2 cannot support the *Cdx1* autoregulatory loop, phenocopying loss of Cdx function and leading to intestinal failure. In contrast, prior work has shown that Cdx2 can support expression from the Cdx1 locus in paraxial mesoderm [21]. These observations provide novel evidence that Cdx members exhibit context-dependent functional specificity in regulating the *Cdx1* promoter.

## Results

**Figure 1:**
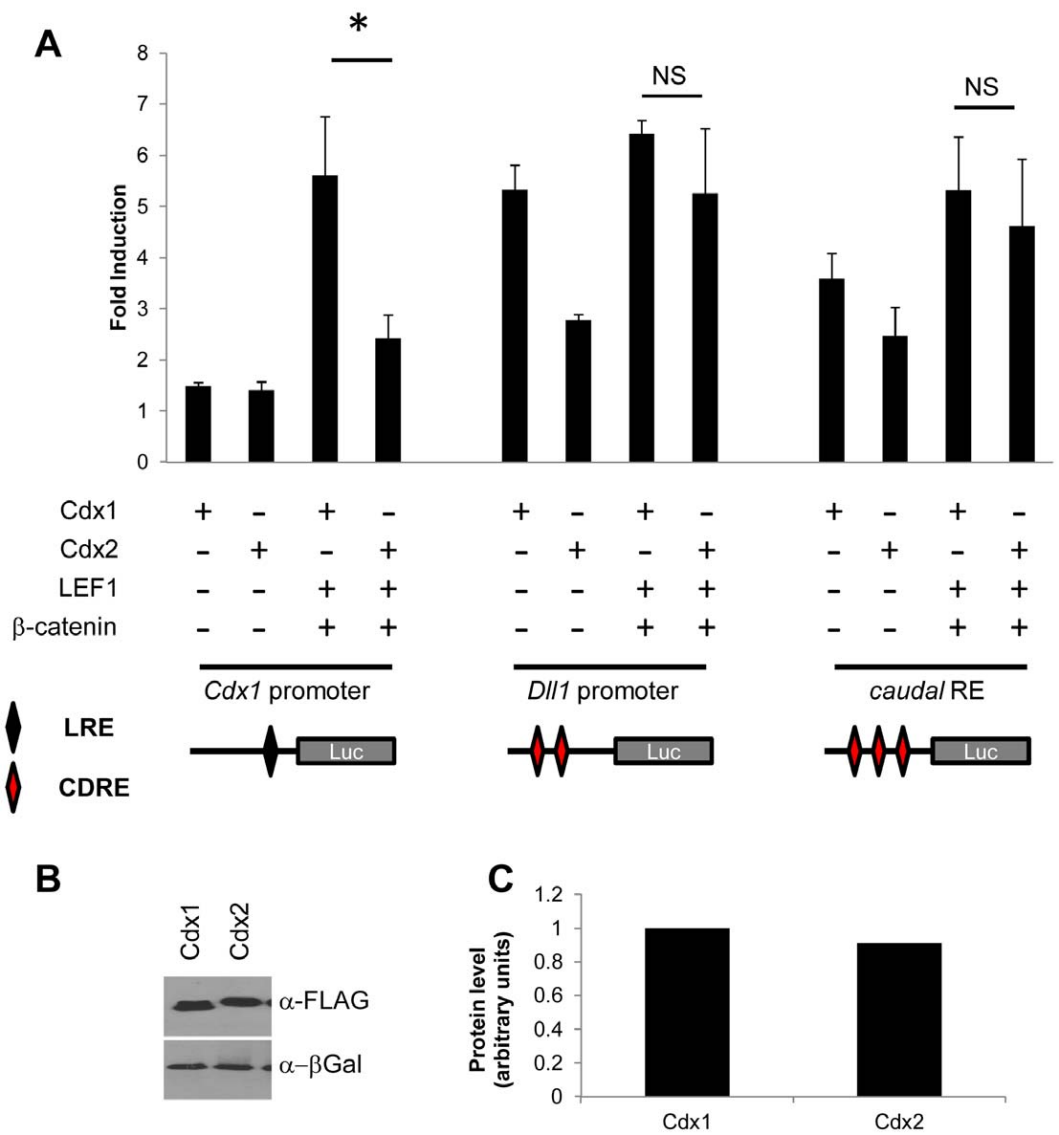
The *Cdx1* promoter is differentially regulated. (A) Luciferase reporter assay in P19 embryonal carcinoma cells with reporters derived from *Cdx1* or *Dll1* promoters or a synthetic Cdx response element (CaudalRE), as noted. Red diamond represent a Cdx response element (CDRE) and black diamond represents a LEF/TCF response element (LRE). Fold induction is shown relative to reporter vector in response to Cdx1 or Cdx2 alone or in combination with LEF1 and β-catenin. Western blot (B) and quantification (C) of Cdx1 and Cdx2 protein using β-galactosidase as a loading control. *P<0.05 by student’s t-test.

Knockout studies have revealed roles for Cdx1 and Cdx2 in anterior-posterior patterning of the endoderm and mesoderm, and in some cases have suggested functional similarity in certain of these programs [17], [18], [21], [22], [29], [30], [31]; however, the specificity of Cdx members on different target genes remains unclear. An auto-regulatory loop comprised of Cdx1 and LEF1 functioning through a LEF/TCF response element in the proximal *Cdx1* promoter has been shown to be critical for *Cdx1* expression [28]. To assess if Cdx2 could function in a comparable manner, we used transfection assays to compare the ability of Cdx1 or Cdx2 to elicit expression from the *Cdx1* promoter alone or with LEF/β-catenin as previously described [28]. We found that, compared to Cdx1, Cdx2 was compromised in its ability to transactivate from the proximal *Cdx1* promoter (Fig. 1A), an outcome that was not due to differences in protein levels (Fig. 1B, 1C). This finding is in contrast to regulation of a promoter derived from the Cdx target gene *Dll1* [22], or a synthetic Cdx response element [32] (Fig. 1A), where both Cdx1 and Cdx2 in combination with LEF/β-catenin induced expression comparably. These findings suggest that Cdx1 and Cdx2 differ in their transcriptional competency on the *Cdx1* promoter.

### Cdx1 N-terminal Sequences Confer Specific Transcriptional Activity

The *Cdx1* autoregulatory loop is thought to be governed by a Cdx1-LEF/TCF complex, with only the latter directly associated with DNA regulatory motifs [28], [33]. To determine if Cdx1 and Cdx2 differentially interact with LEF/TCF members, we compared their association with LEF1 or TCF4 (also known as TCF7l2), the predominant LEF/TCF family member in the intestine [34], [35]. We found that both Cdx1 and Cdx2 interacted comparably with either LEF1 or TCF4 (Fig. S1 and data not shown). These results suggested that differential affinity between Cdx and LEF/TCF proteins does not underlie the disparate transactivation competency observed between Cdx1 and Cdx2 on the *Cdx1* promoter.

**Figure 2:**
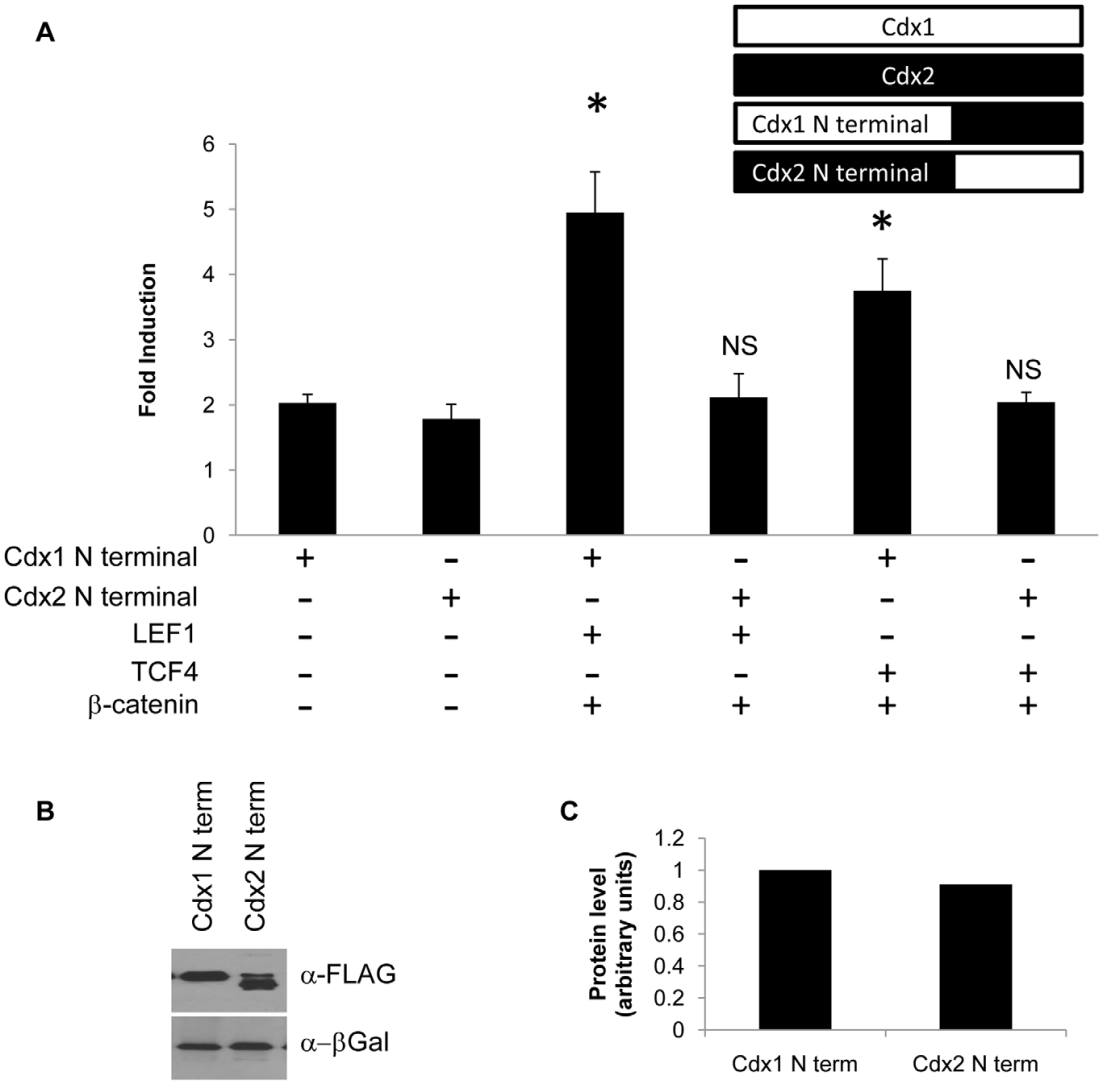
Differential regulation by N-terminal Cdx transcriptional activation sequences. (A) Regulation of a *Cdx1* promoter reporter vector in P19 embryonal carcinoma cells. Fold induction is shown relative to reporter vector alone in response to fusion proteins harboring the Cdx1 N terminal and Cdx2 homeodomain (Cdx1 N terminal) or the converse construct (Cdx2 N terminal). Transfections were conducted in combination with either LEF1 and β-catenin or TCF4 and β-catenin. Western blot (B) and quantification (C) of Cdx1 N-terminal and Cdx2 N-terminal proteins using β-galactosidase as a loading control. *P<0.05 by student’s t-test.

**Figure 3:**
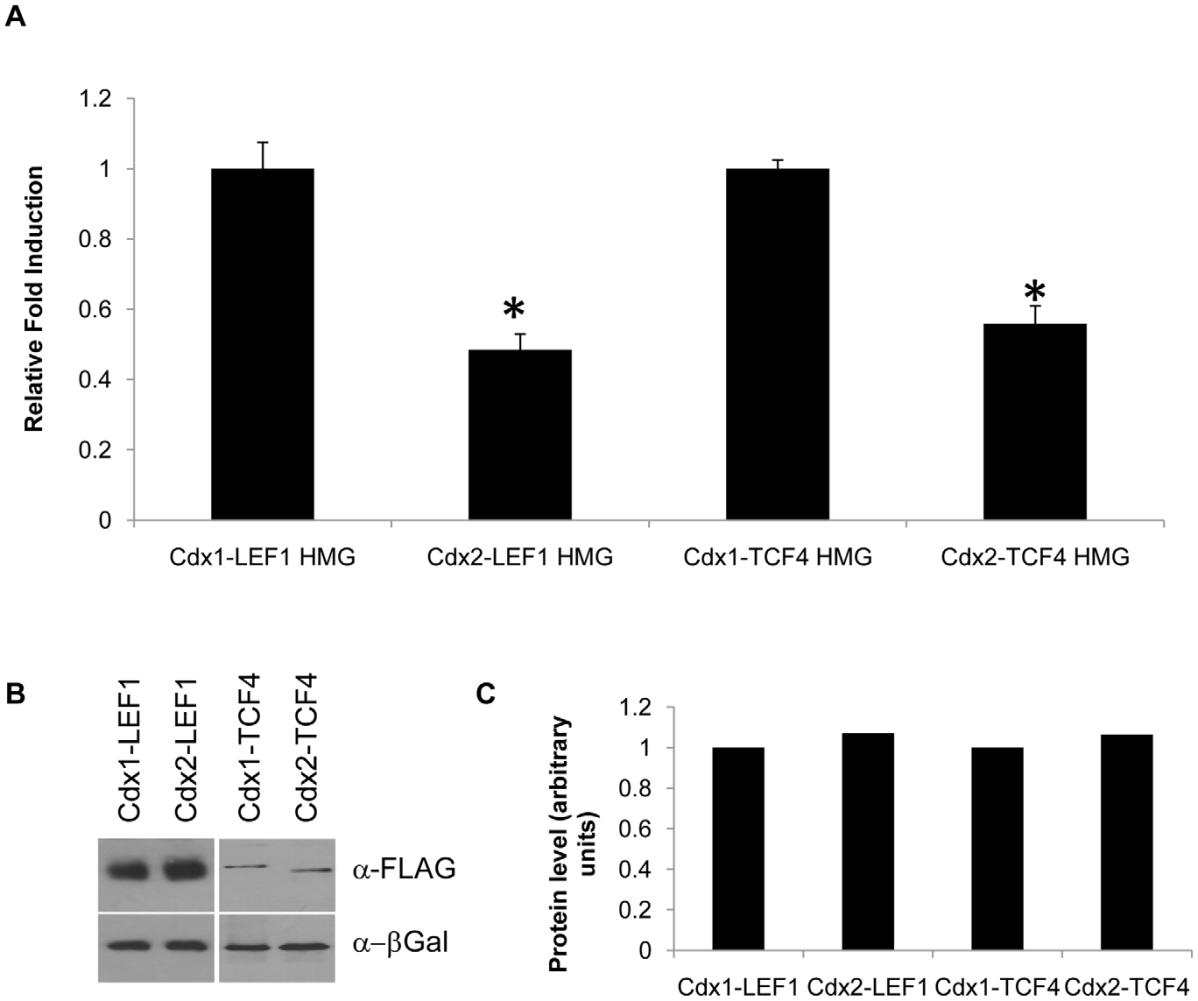
Cdx1 and Cdx2 differ in their N-terminal activation domains. (A) Cdx1 promoter regulation in P19 embryonal carcinoma cells. Fold induction is shown relative to reporter vector alone, in response to chimeric proteins harboring the Cdx1 N terminus fused to LEF1 or TCF4 DNA binding domains (Cdx1-LEF1 HMG, Cdx1-TCF4 HMG, respectively) or analogous constructs using the Cdx2 N terminus (Cdx2-LEF1 HMG, Cdx2-TCF4 HMG). Western blot (B) and quantification (C) of Cdx1 and Cdx2 fusion proteins using β-galactosidase as a loading control. *P<0.05 by student’s t-test compared to Cdx1-HMG protein.

To begin to assess the basis for the differential transcriptional potency between Cdx1 and Cdx2, chimeric proteins in which the N-terminal sequences of each family member were exchanged were assessed for regulation of the *Cdx1* promoter. This analysis revealed that Cdx1, but not Cdx2, N-terminal sequences were more potent at inducing transcription from this promoter (Fig. 2A). In addition, chimeric proteins harboring the N-terminal of Cdx1 fused to the DNA binding HMG domain of LEF1 or TCF4 were also more effective at eliciting expression from the *Cdx1* promoter than the comparable Cdx2 derivatives (Fig. 3A). These differences were not due to variance in protein levels (Fig. 2B, 2C, 3B, 3C), but rather suggest that the basis for the observed functional differences between Cdx1 and Cdx2 lies in properties inherent to their N-terminal transactivation sequences.

### Cdx2 Cannot Support Cdx1 Autoregulation in vivo

As described above, and by others, Cdx members exhibit different transactivation potential on a number of target genes in tissue culture models [24], [25], [26], [27]. It is, however, unclear if these differences hold *in vivo*. To investigate this, we used a previously described knock-in allele in which the *Cdx2* open reading frame was inserted into the *Cdx1* locus (hereafter designated *Cdx1^2ki/2ki^*), resulting in replacement of Cdx1 with Cdx2 [21]. We then crossed this line with the conditional *Cdx2^f/f^ villin* Cre ER^T^ line, and mated *Cdx1^2ki/2ki^Cdx2^f/f^ villin* Cre ER^T^ males with *Cdx1^2ki/2ki^ Cdx2^f/f^* females, deleting *Cdx2* in the intestinal epithelium either at E13.5 or in the adult as previously described [7], [23]. Cre-positive offspring (referred to as *Cdx1^2ki/2ki^Cdx2^−/−^*) were anticipated to lack expression of *Cdx2* from the endogenous allele [7] and to express *Cdx2* under the regulation of the *Cdx1* promoter. Littermate controls (*Cdx1^2ki/2ki^*) were anticipated to express *Cdx2* from both the endogenous and knock-in alleles.

**Figure 4:**
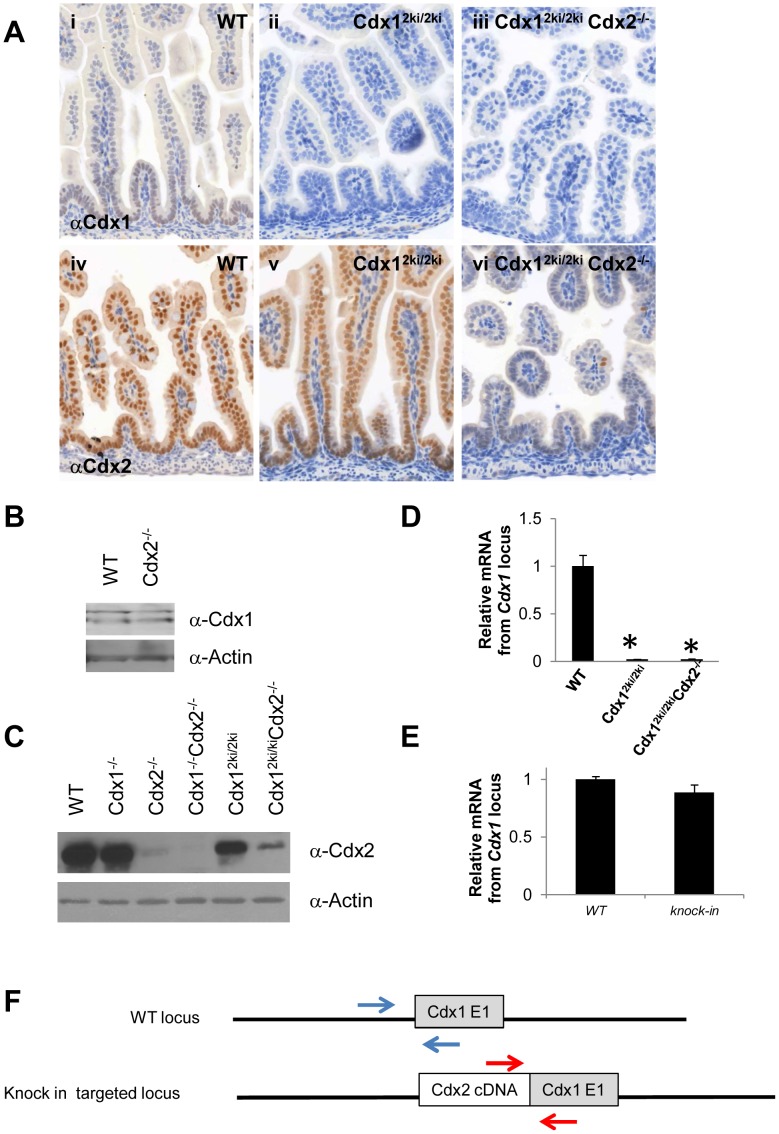
Cdx2 does not support expression from the *Cdx1* promoter in the small intestine. (A) Immunohistochemistry for Cdx1 (i-iii) or Cdx2 (iv-vi) in the small intestine of E18.5 WT (i, iv), *Cdx1^2ki/2ki^* (ii, v) or *Cdx1^2ki/2ki^Cdx2^−/−^* (iii, vi) animals. Western blot analysis for Cdx1 (B) or Cdx2 (C) and actin loading controls from small intestine. (D) qPCR analysis for transcripts from the *Cdx1* locus in knock-in animals (red arrows in F) relative to WT (blue arrows in F). (E) qPCR analysis for WT (blue arrows in F) and knock-in (red arrows in F) transcripts from the *Cdx1* locus heterozygous animals. *P<0.05 by student’s t-test. (F) Schematic representation of the wild type and targeted *Cdx1* allele with primer sets for measuring WT (blue) and knock in (red) transcripts.

As expected, neither *Cdx1^2ki/2ki^Cdx2^−/−^* nor *Cdx1^2ki/2ki^* mice exhibited Cdx1 expression (Fig. 4A i–iii), consistent with prior work [21]. *Cdx1^2ki/2ki^* mice displayed robust Cdx2 expression along the entire crypt-villus axis, comparable to wild type controls (Fig. 4A; panels iv and v). In marked contrast, *Cdx1^2ki/2ki^ Cdx2^−/−^* offspring had very low levels of Cdx2, with more robust staining at the base of the crypts and tapering off towards the villus tip (Fig. 4A vi). This is similar to (but much weaker than) the normal pattern of expression of Cdx1 (Fig. 4A, panel 1) [12], [36]. Consistent with these observations, western blot analysis showed markedly decreased expression of Cdx2 in *Cdx1^2ki/2ki^ Cdx2^−/−^* animals compared to controls (Fig. 4C).

**Figure 5:**
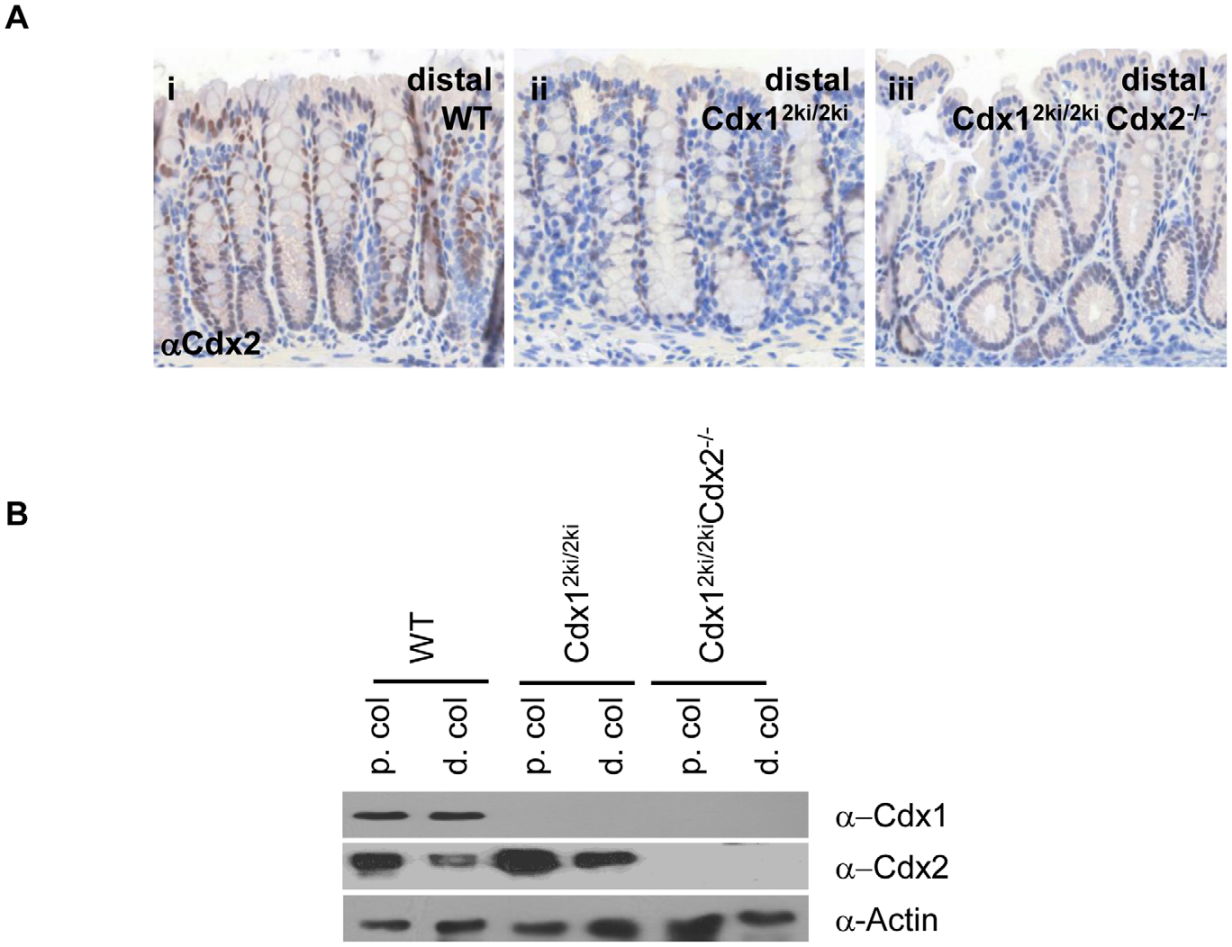
Cdx2 cannot drive expression from the *Cdx1* promoter in the adult colon. (A) Cdx2 immunohistochemistry and (B) Western blot analysis of Cdx2 in proximal and distal colon from WT (i), *Cdx1^2ki/2ki^* (ii) and *Cdx1^2ki/2ki^Cdx2^−/−^* (iii) mice.

The above data suggest that *Cdx2* is not efficiently expressed from the *Cdx1* promoter in the small intestine. To investigate this, we examined transcripts produced from the *Cdx1* promoter by qPCR. The small intestine of *Cdx1^2ki/2ki^* mice exhibited a strong reduction in transcripts derived from the *Cdx1* promoter (Fig. 4D), suggesting a failure in transcription. This was not due to unforeseen effects mediated by the knock-in *per se*, as *Cdx1^+/2ki^* mice produced comparable levels of both wild-type and knock-in messages (Fig. 4E). It is also notable that loss of Cdx2 in *Cdx1^2ki/2ki^Cdx2^−/−^* mice had no impact on expression from the *Cdx1* promoter (Fig. 4D), consistent with the finding that loss of Cdx2 did not affect Cdx1 levels (Fig. 4B). A similar relationship was seen in the adult colon, as assessed by both immunohistochemistry (Fig. 5A) and western blot analysis (Fig. 5B), although there appeared to be a modest increase in Cdx2 levels in *Cdx1^2ki/2ki^* mice compared to wild-type controls, suggestive of compensatory mechanisms. Taken together, these findings indicate that Cdx1, but not Cdx2, is necessary for transcription from the *Cdx1* promoter in the intestine.

### Expression of Cdx2 from the Cdx1 Locus does not Support Intestinal Development or Homeostasis

Previously, we demonstrated that loss of Cdx2 in the small intestine at E13.5 leads to transformation of the intestinal epithelium to a partial glandular stomach identity. Cdx1 has no discernable role in this process [7], [13]. Immunohistochemistry and qPCR data indicated residual *Cdx2* transcripts were produced from the *Cdx1* promoter in *Cdx1^2ki/2ki^Cdx2^−/−^* mice. We therefore assessed the small intestine of *Cdx1^2ki/2ki^Cdx2^−/−^* mice to determine if this residual Cdx2 protein could support intestinal patterning.

**Figure 6:**
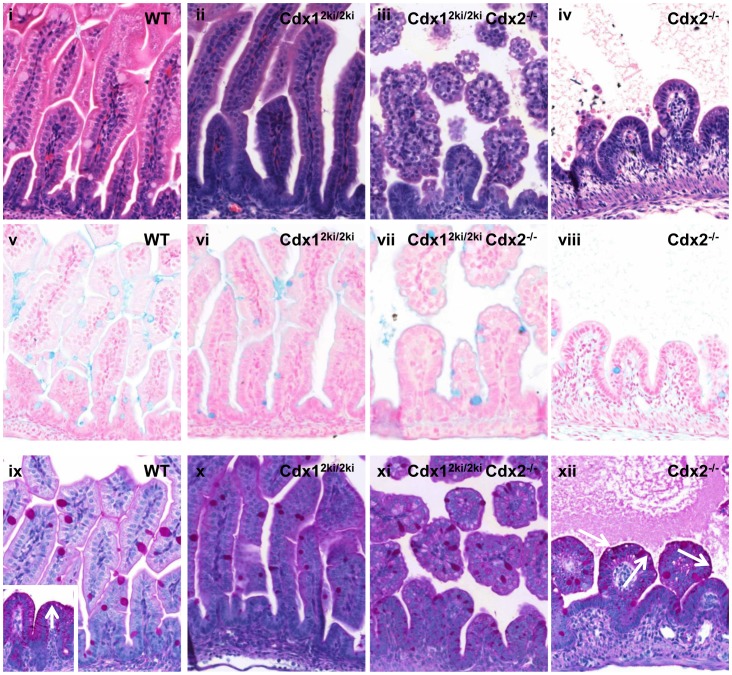
The Cdx2 knock in allele cannot complement loss of endogenous Cdx2. Dams bearing *Cdx1^2ki/2ki^*-*Cdx2^f/f^ villin* cre-ER^T^ mice or non-transgenic controls (designated WT) were treated with 5 mg of tamoxifen at embryonic day (E)13.5. Intestinal tracts were harvested at E18.5, fixed, sectioned and stained with hematoxylin and eosin (i-iv), Alcian Blue (v-viii) or Periodic Acid Schiff (PAS) (ix-xii). Shown are representative sections of small intestine from *WT* (i, v, ix), *Cdx1^2ki/2ki^* (ii, vi, x), *Cdx1^2ki/2ki^Cdx2^−/−^* (iii, vii, xi) and *Cdx2^−/−^* (iv, viii, xii) offspring. WT pyloric stomach is in the inset in ix. White arrows in panel xii indicate apical PAS staining, typical of pyloric stomach (inset in ix).

*Cdx1^2ki/2ki^* mice appeared normal (data not shown) and histological analysis revealed no apparent abnormalities in these animals (Fig. 6; panels i and ii). In contrast, the duodenum of *Cdx1^2ki/2ki^ Cdx2^−/−^* mice exhibited a disordered epithelium, with shortened villi and vacuolated cells (Fig. 6; panel iii), reminiscent of the outcome of Cdx2 loss at E13.5 [7] (Fig. 6, panel iv). As previously described [7], excision mediated by the *villin* Cre ER^T^ transgene during development of the large intestine was not sufficient to warrant further study [7].

Periodic Acid-Schiff (PAS) stains mucins in the apical edge of glandular stomach, as well as in Goblet cells of the intestine, while Alcian Blue stains mucins only in Goblet cells of the intestinal epithelium and not the stomach [37]. *Cdx2^−/−^* mice exhibit ectopic PAS staining along the apical edge of the aberrant villi, indicative of a transformation to pyloric stomach (Fig. 6 panel xii), as well as supernumerary intestinal goblet cells due to aberrant Notch signaling [7], [22]. In contrast, neither *Cdx1^2ki/2ik^* nor *Cdx1^2ki/2ki^Cdx2^−/−^* mice exhibited ectopic PAS or Alcian Blue staining, although *Cdx1^2ki/2ki^ Cdx2^−/−^* mice appeared to have more Goblet cells (Fig. 6 panels vi, vii, x, xi and data not shown). These results suggest that expression of *Cdx2* under *Cdx1* regulatory elements mice partially supports Cdx2-dependent function in the small intestine.

**Figure 7:**
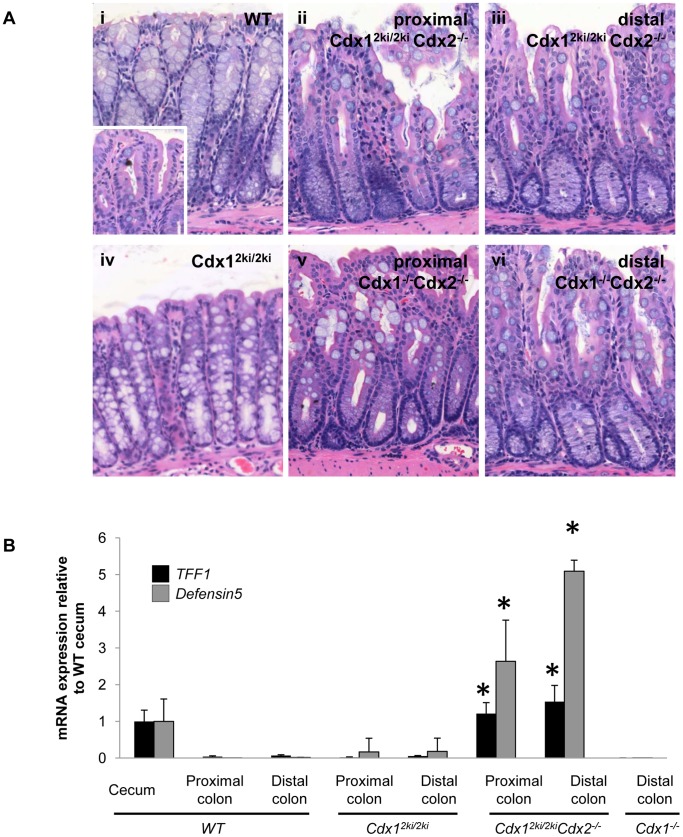
The Cdx2 knock in allele cannot support colon homeostasis. (A) Hematoxylin and eosin staining of control adult cecum (i), *Cdx1^2ki/2ki^* colon (iv) and proximal and distal colons from *Cdx1^2ki/2ki^Cdx2^−/−^* (ii, iii) and *Cdx1^−/−^Cdx2^−/−^* (v, vi) mice. Note the semblance of the proximal and distal colon in *Cdx1^2ki/2ki^Cdx2^−/−^* and *Cdx1^−/−^Cdx2^−/−^* offspring to wild-type cecum. (B) qPCR analysis for the cecum-enriched transcripts *TFF1* and *Defensin5*. *P<0.05 by student’s t-test compared to wild-type controls.

In wild type mice, Cdx1 protein levels are maximal in the distal colon, while Cdx2 peaks in the proximal cecum and diminishes in either direction [2], [12], [36], [38], [39]. Cdx members are critical for differentiation and homeostasis of the entire adult intestinal tract, including the large intestine [8], [23], [40]. In this regard, combined loss of Cdx1 and Cdx2 results in anteriorization of the distal colon to a cecal character [23], revealing a role for Cdx1 in the colon that is seen only with concomitant loss of Cdx2. To test if Cdx2 could drive sufficient expression from the *Cdx1* locus to fulfill Cdx function in the distal intestine, we examined the colon in *Cdx1^2ki/2ki^Cdx2^−/−^* offspring. While *Cdx1^2ki/2ki^* colons appeared normal, *Cdx1^2ki/2ki^Cdx2^−/−^* colons exhibited scalloped glands in the place of the typical flattened colonic epithelium, similar to those seen in *Cdx1^−/−^Cdx2^−/−^* offspring [23] (Fig. 7A), and suggestive of a conversion to a cecum-like phenotype. This is consistent with gain of expression of the cecal-enriched genes *Defensin5* and *TFF1* [41] (Fig. 7B). These data suggest a marked loss of Cdx function in the distal intestine [23], consistent with an inability of Cdx2 to drive expression from the *Cdx1* locus in the intestinal tract *in vivo*.

## Discussion

Recent studies have revealed critical functions for Cdx2 in diverse processes, including patterning the endoderm [7], [8], [9], [23], intestinal differentiation [9], [22], [40] and axial elongation [17], [18]. Despite divergence outside of their homeodomains, Cdx members exhibit significant functional overlap, as exemplified by the ability of Cdx2 to complement Cdx1 mutants in vertebral patterning [21]. However, the functional relatedness of Cdx members is less clear as regards the intestinal tract. To this end, we examined regulation of *Cdx1*, a known direct Cdx target gene expressed in the intestine [12], [28]. We found that Cdx2 was unable to drive expression from the *Cdx1* promoter *in vitro*, or *in vivo*. Furthermore, we found that Cdx2 could not be expressed from the *Cdx1* locus at levels that suffice to support Cdx-dependent roles in the gastrointestinal tract. This represents the first *in vivo* demonstration of functional specificity between these family members.

### Specific Regulation of the Cdx1 Promoter

We showed that Cdx2 is less efficient at transactivation from the *Cdx1* promoter relative to Cdx1 using tissue culture based models. This finding was recapitulated in *Cdx1^2ki/2ki^Cdx2^−/−^* mice, which have greatly diminished levels of Cdx2 and phenocopy intestinal Cdx loss of function models [22], [23], [31], [40]. The loss of Cdx2 arises due to a failure of transcription at the *Cdx1* locus, evident from transcriptional readout at the *Cdx1* locus by qPCR. These findings illustrate that Cdx2 is less potent than Cdx1 in regulating the *Cdx1* promoter throughout the extent of the intestinal tract and during development and in the adult.

### N-terminal Sequences Convey Differential Cdx Function

Cdx1 and Cdx2 have divergent N terminal sequences which harbor poorly defined transactivation functions [2], [42], [43]. Consistent with previous work [28], [33], we found that Cdx1 could direct transcription from its own promoter in concert with LEF/TCF in tissue culture models, but that this effect was not efficiently recapitulated by Cdx2. Although this auto-regulation relies on association with a LEF/TCF member bound to the proximal *Cdx1* promoter [28], differential association between LEF/TCF and Cdx1 or Cdx2 did not appear to underline the specificity of *Cdx1* regulation. Rather this specificity appears to reside in the divergent N-terminal sequences, suggesting that the transactivation domain of Cdx1 differentially interacts with a transcriptional co-regulator(s) needed for transcription from the *Cdx1* locus. Because Cdx members interact physically with LEF/TCF members through conserved homeodomain sequences, it is also unlikely that differential expression of LEF/TCF members underlies this effect. In this regard, recent work has suggested that Cdx2 is capable of association with a number of intestinal transcription factors in a manner that reflects the differentiation state of the cell [40], and it is possible that such a co-regulator may underlie Cdx-specific transcriptional regulation.

The paradigm of Cdx specificity may extend to other target genes. For example, the Cdx target *Dll1* is regulated by Cdx1 and Cdx2 in the presomitic mesoderm, but is not impacted by Cdx1 loss in the intestine [22]. Cdx1 and Cdx2 also exhibited different levels of transcriptional potency on the *Dll1* promoter in P19 embryocarcinoma cells in the current study. However, *Dll1* has also been shown to be a Wnt target [44], [45] and similar levels of induction were seen with Cdx1 and Cdx2 in conjunction with LEF/β-catenin (Fig. 1A). This is again consistent with modulation of Cdx activity by collaborative partners [9], [17].

### Functional Specificity of Cdx Members

Substantial data suggests functional overlap between Cdx members in diverse ontogenic programs. For example, the phenotype of single versus compound Cdx loss suggests overlap as regards vertebral patterning, axial elongation and neural tube closure [13], [14], [15], [17], [19]. This is also consistent with gene substitution approaches which have shown that Cdx2 can fully complement Cdx1 loss in vertebral patterning [21]. Indeed, this latter observation used the same *Cdx1^2ki/2ki^* line employed in the current study, clearly illustrating that Cdx2 can fulfill *Cdx1* autoregulation in the mesoderm, but not in the intestine, and thus impacts on this target gene in a context specific manner.

In contrast to the above observations, work from tissue culture models presents conflicting evidence regarding specificity of Cdx1 and Cdx2 in regulation of intestinal genes [24], [25], [26], [27], [46], [47]. Additional data from mouse models [7], [9], [10], [23], [40] show that loss of Cdx1 does not appear to play any role in patterning [7], [13], [48] or differentiation [22] of the small intestine, even in the absence of Cdx2. In contrast, functional overlap between Cdx1 and Cdx2 has been suggested in the adult GI tract, where deletion of Cdx1 results in an exacerbation of intestinal failure associated with Cdx2 loss and the appearance of a novel colon phenotype [23], [40]. Finally, gene profiling of *Cdx2^−/−^* and *Cdx1^−/−^Cdx2^−/−^* mutants from *villin-*Cre ER^T^ conditional mice suggest that there are both common and specific intestinal Cdx target genes [8].

Transgenic models also suggest differential function between Cdx1 and Cdx2. For example, overexpression of Cdx2 in the gut epithelium is lethal [49] while overexpression of Cdx1 does not cause any overt phenotype [48], [50]. However, these models resulted in abnormal Cdx expression, which may result in non-physiological impact. Similarly, misexpression of Cdx1 or Cdx2 in the stomach, which is normally devoid of Cdx, evokes slightly different phenotypes [51]. However, it is unknown if these outcomes reflect specific function or are due to differences in expression levels. Further characterization of Cdx1 and Cdx2 target genes and binding partners will be needed to better understand their molecular mechanisms of action.

## Experimental Procedures

### Mice

*Cdx1^−/−^*, *Cdx2^f/f^*, *Cdx1^2ki/2ki^* and *villin-*Cre ER^T^ mice have been previously described [13], [17], [21], [52], [53]. *Villin-*Cre ER^T^ *-*mediated deletion of Cdx2 was initiated by Tamoxifen administration at embryonic (E)13.5 or at 6 weeks of age as previously described [7], [23]. At appropriate times, animals were sacrificed by cervical dislocation and gastrointestinal tracts were harvested at E18.5 or 6 days post-treatment in adults. Tamoxifen-treated non-transgenic littermates were used as controls in all instances. In all cases, experiments were repeated with a minimum of 3 different animals. The work described in this study was approved by the Animal Care and Veterinary Service of the University of Ottawa in accordance with the guidelines of the Canadian Council for Animal Care.

### Histology and Western Blot Analysis

E18.5 intestinal tracts were processed for histological and immunohistochemical staining as previously described [7]. Slides were mounted using Permount (Fisher) and images captured using a Zeiss Mirax Midi Scanner (Zeiss). Protein was harvested using 500 µL of solubilizer buffer (8 M urea, 4% CHAPS, 2 mM DTT, protease inhibitor cocktail (Chemicon)). Lysates were sonicated for 30 s at 30% output using a Branson sonifier 450, lysates cleared by centrifugation at 14,000 g for 20 minutes at 4°C and proteins quantified using the Bradford method [54]. Western blots were performed as previously described [7].

### Plasmid Constructs

The glutathione S-transferase (GST)-Cdx1 and GST-Cdx2 fusion proteins have been described previously [28]. GST-LEF1 and GST-TCF7l2 contstructs were derived by subcloning appropriate open reading frames into pGEX4T-1. A FLAG-tagged TCF7l2 expression vector was generated using plasmid number 11031 (p043 mTCF-4B) from Addgene [55]. The LEF1-HA, Cdx1 and Cdx2 expression vectors and *Cdx1*-luciferase reporter vectors have been previously described [28].

### GST Fusion Proteins

BL-21 bacteria were transformed with either empty GST expression plasmid, GST-Cdx1, GST-Cdx2, GST-LEF1 or GST-TCF7l2 fusion constructs. Cultures were grown to an OD_600_ of 0.5, treated with 0.5 mM IPTG (Bioshop), and cultured for a further 3 hours. Cells were then pelleted, resuspended in PBS containing 1% Triton X-100, 1 mM DTT and protease inhibitors (1 µg/mL aprotinin, 1 µg/mL leupeptin, 1 µg/mL pepstatin A, 1 mM PMSF; Sigma) and lysed by sonication using a Branson Sonifier 450. Cell debris was cleared by centrifugation at 10,000 g. Binding and subsequent washing of glutathione-agarose beads (BD Biosciences) was carried out as per the manufacturer’s recommendations. Beads were analyzed for effective binding and equal loading by Coomassie staining prior to use.

### Tissue Culture and Transfection

P19 cells were grown under standard conditions. Transfections were carried out using the calcium phosphate precipitation method. For promoter analysis, P19 cells in 6 well plates were transfected with 1 µg reporter vector, varying amounts of expression vectors and 200 ng of β-galactosidase expression vector, to a total of 3 ug of DNA per well. Cells were harvested 48 hours post-transfection and the lysates processed and analyzed using the Promega Luciferase Assay System according to the manufacturer’s instructions. β-galactosidase activity was assayed using the chlorophenolred-ß-D-galactopyranoside (CPRG) assay system (Calbiochem) and used to correct for transfection efficiency.

### Protein-protein Interaction Assays

*In vitro* protein-protein interaction assays were conducted as previously described [28] using the Quick-coupled transcription and translation kit (Promega) according to manufacturer’s recommendations. For each assay, 5 µg of GST fusion protein and 5 µL of translation reaction were used. Inputs shown represent 5% of the total protein used for immunoprecipitation.

### Quantitative Reverse-transcriptase Polymerase Chain Reaction (qPCR)

RNA was extracted from E18.5 small intestine using Trizol reagent (Invitrogen) and used to generate cDNA by standard procedures. cDNA was subsequently amplified by qPCR using oligonucleotides specific for the *Cdx2* replacement allele, wild-type *Cdx1* or *β-actin.* qPCR was performed using the MX3005P cycler (Agilent Technologies) with SsoFast EvaGreen Supermix (qPCR, BioRad), according to the manufacturer’s recommendations. Results were analyzed using the 2^−ΔΔCt^ method [56], normalized for *β-actin,* with the dissociation curve considered for the specificity of each amplicon. Results reflect the mean of three independent biological samples. The primers used were: for wild type F- 5′GGGCCCAGCATGCGCGG3’ and R- 5′CGCGAAGTCGGGGTACTGCG3’; for knock in F- 5′GCAGTCGCTGGTCGTCGG3’ and R- 5′GGAGGACTGACAAAGTTCTGCGG3’. Other primer sequences are available upon request.

## Supporting Information

Figure S1 **Cdx1 and Cdx2 are comparable in binding TCF7l2 and LEF1** ***in vitro*** **.** Cdx1 and Cdx2 (A) or TCF7l2 and LEF1 (B) were transcribed and translated in vitro in the presence of ^35^S-methionine and pulled down with GST-Cdx1 and GST-Cdx2 (B) or GST-TCF7l2 and GST-LEF1 (A). Inputs represent 5%. Note that Cdx1 and Cdx2 both bind to TCF7l2 and LEF1.
(TIF)
