# Supplementary figures and images for: Cdx1 and Cdx2 Exhibit Transcriptional Specificity in the Intestine
Source: PLoS One. 2013 Jan 30;8(1):e54757. doi: 10.1371/journal.pone.0054757 (PMC3559873; doi:10.1371/journal.pone.0054757)

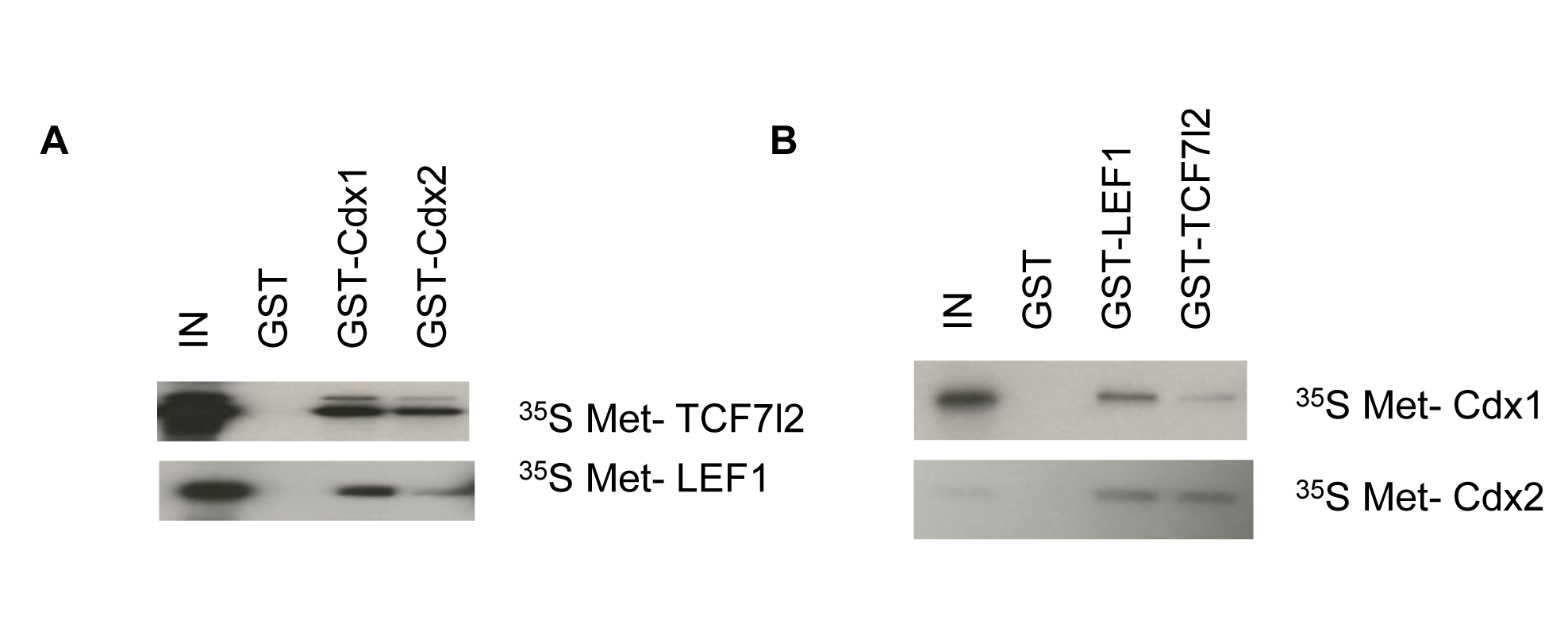

Supplement: Figure S1 — Cdx1 and Cdx2 are comparable in binding TCF7l2 and LEF1 in vitro . Cdx1 and Cdx2 (A) or TCF7l2 and LEF1 (B) were transcribed and translated in vitro in the presence of 35S-methionine and pulled down with GST-Cdx1 and GST-Cdx2 (B) or GST-TCF7l2 and GST-LEF1 (A). Inputs represent 5%. Note that Cdx1 and Cdx2 both bind to TCF7l2 and LEF1. (TIF) [file pone.0054757.s001.tif]
